# Organizational Characteristics of Medical-Legal Partnerships in HIV Care: Exploring Challenges and Opportunities through a Mixed Methods Study

**DOI:** 10.1017/jme.2025.10142

**Published:** 2025

**Authors:** Allen Partono, Miguel Munoz-Laboy, Ashley French, Robin Davison, Xiang Zhu, Omar Martinez

**Affiliations:** 1 University of Central Florida, College of Medicine, Orlando, FL, USA; 2 Stony Book University, School of Social Welfare, Stony Brook, NY, USA

**Keywords:** Medical-Legal Partnership, HIV Care Continuum, Mixed Methods, Health-Harming Legal Needs, People with HIV

## Abstract

People with HIV (PWH) often encounter health-harming legal needs that impede their access to care, including structural issues such as racism, discrimination, unstable housing, and stigma. Medical-Legal Partnerships (MLPs) have emerged as a promising strategy to address these challenges within HIV care settings. This study aimed to identify the characteristics and strategies of MLPs that are most effective in improving HIV care continuum outcomes. A mixed-methods analysis was conducted utilizing data from a cross-sectional survey of 60 providers in MLPs. Categorical features of MLPs, such as the personnel responsible for screening for health-harming legal needs (HHLN), the organizational structure (community-based vs. institutional), and the delivery of legal services, were examined. A multiple, variable linear regression analysis was conducted to explore the association between these variables and outcomes. Community health organizations were found to be associated with a greater number of patients achieving suppressed HIV viral load. Additionally, a higher number of on-site services were positively correlated with a greater percentage of PWH achieving decreased viral load and completing follow-up appointments. Findings underscore the significance of comprehensive care approaches within MLPs for enhancing positive patient outcomes in HIV care settings.

## Background

Human immunodeficiency virus (HIV) remains a major public health concern in the United States, disproportionately affecting racial, ethnic, and sexual and gender minority groups. For instance, African Americans make up 37% (11,900) of new diagnoses despite being only 12% of the US population.^1^ Moreover, African Americans with HIV, particularly women, often experience worse mental health outcomes due to microaggressions and discrimination.^2^ Additionally, of the 31,800 estimated new HIV infections in the US in 2022, 33% (10,500) were among Hispanic/Latino people.^3^ The Centers for Disease Control and Prevention (CDC) reported that 67% of HIV infections in 2022 were attributable to male-to-male sexual contact, highlighting the disproportionate impact on sexual minority men.^4^ Studies also indicate that sexual minority men face disproportionately high rates of undiagnosed HIV.^5^

The HIV care continuum comprises various stages, including diagnosis, linkage to anti-retroviral treatment (ART) services, retention in care, ART adherence, and viral suppression.^6^ Despite advancements in the HIV care continuum, challenges remain in connecting and retaining people with HIV (PWH) in care, with only 76% of diagnosed individuals receiving some HIV care, 54% retained in care, and 65% achieving viral suppression.^7^ Studies have indicated that this gap in care is particularly pronounced among certain groups, including non-Hispanic Black and African American individuals.^8^ Structural barriers, including insurance, stigma, housing insecurity, medical mistrust, employment, and discrimination further impede access to care for PWH.^9^ These health-harming legal needs (HHLN) tend to interfere with the ability of PWH to properly manage their care. For example, one report highlighted that a substantial number of PWH experienced discrimination across multiple areas of life including employment termination, denial of medical care, loss of insurance coverage, and housing eviction, which adversely affected their ability to manage their health.^10^ Despite the importance of addressing these needs, many medical providers lack the training and resources to do so effectively.^11^

Over the past two decades, researchers have dedicated efforts to developing and refining Medical-Legal Partnerships (MLPs) as a means of enhancing health outcomes and addressing HHLN, particularly among systematically and structurally excluded populations.^12^ An MLP represents an innovative integration of legal services and advocacy within healthcare delivery systems, providing a holistic approach to addressing the complex needs of patients, including those with HIV.^13^ Despite the growing recognition of MLPs’ potential, their implementation and utilization within healthcare systems vary widely. Consequently, there remains a notable gap in the literature regarding the specific components of MLPs that effectively contribute to retaining PWH within the HIV care continuum. This knowledge gap underscores the need for comprehensive research to identify and elucidate the key features and strategies of MLPs that maximize their impact on patient retention and healthcare outcomes in the context of HIV care.

The primary aim of this study is to conduct a comprehensive analysis of the implementation strategies and organizational characteristics of MLPs that have demonstrated significant value in enhancing patient outcomes for PWH. Additionally, we seek to gain insights into the challenges and benefits associated with the utilization of MLPs to support PWH along the HIV care continuum. To achieve this objective, we employ the Health Equity Implementation Framework^14^ as a guiding conceptual model. This framework provides a structured approach to understanding how the implementation of healthcare innovations, such as MLPs, is influenced by various factors at individual, organizational, and societal levels. The Health Equity Implementation Framework delineates the multifaceted interplay between individual patients, healthcare providers, organizational structures, and broader sociocultural and economic influences. By examining these dynamic interactions, we aim to elucidate the mechanisms through which MLPs impact patient care and outcomes within the context of HIV treatment and management. Key components of MLPs that are analyzed within this framework include the professional roles responsible for conducting screenings for health-harming legal needs, the organizational structure and culture within healthcare settings, and the availability of comprehensive services to address both legal and social determinants of health.

Despite the growing recognition of the importance of MLPs in addressing health disparities and promoting health equity, there remains a paucity of literature examining the specific organizational characteristics of MLPs that contribute to improved healthcare outcomes, particularly within the realm of HIV care.^14^ Therefore, this study endeavors to fill this gap by investigating select organizational features of MLPs that cater to PWH. By identifying and evaluating these key components, we aim to discern which aspects of MLP implementation are most effective in enhancing the overall quality of care and health outcomes for PWH, thereby informing future efforts to optimize the delivery of HIV care services through MLPs.

## Methods

### Sample

We undertook a comprehensive mixed-method analysis to examine the intricate relationship between both quantitative and categorical variables and their impact on HIV viral load. Our study encompassed 60 cross-sectional survey responses drawn from a diverse pool of participants, including clinicians, social and behavioral health service providers, and administrators actively engaged in MLPs across the United States. To ensure the robustness and relevance of our sample, strict inclusion criteria were applied, specifically targeting organizations catering to patient populations with over 50% of patients with HIV.

Engagement with MLP service providers was fostered through proactive outreach efforts, including participation in the annual MLP summit and targeted communication in the three months preceding the event. Furthermore, we tapped into the rich resources of the National Center of Medical-Legal Partnership (NCMLP) and Ryan White Clinic databases, in addition to establishing collaboration with established HIV MLP partners such as Whitman-Walker Health and the AIDS Law Project of Pennsylvania. The NCMLP database, housing information on existing MLPs, their geographic locations, and contact details, served as a vital resource. At the time of our study, the database listed a total of 294 MLPs spanning 41 states, each entry providing contact information for two to three key stakeholders. This extensive network facilitated our outreach efforts and enriched the diversity of perspectives captured in our survey responses. Furthermore, we developed online recruitment platforms that included the creation of comprehensive recruitment materials, curated by a scientific advisory board to emphasize the study’s importance. We utilized social media accounts to disseminate information about the study through tweets, engaging prominent MLPs to amplify the message.

Clear communication regarding the survey’s objectives and data utilization was provided to all participants. Importantly, stringent measures were implemented to safeguard the confidentiality of respondents and their patients, with the survey being conducted online and devoid of any collection of identifiable information. Recognizing the valuable contribution of study participants, we implemented a modest incentive structure, offering a US$30 gift card to respondents upon completion of the survey. The study secured approval from the Temple University Institutional Review Board.

### Outcome

The outcome variables of the percentage of patients with a suppressed HIV viral load and the percentage of patients retained in care are both quantitative. We selected these outcomes based on the HIV care continuum due to their established link to long-term morbidity and mortality. In our study, the independent variables encompassed both quantitative and categorical aspects. The quantitative variables comprised the number of on-site services available, and the number provided via referral. Categorical variables included the type of healthcare organization, the role of the individual conducting the screening for HHLN (clinician, social or behavioral health worker, law practitioner, or administrator), and whether legal services were provided on-site or through referral. These variables represent various internal organizational factors and policies. We categorized healthcare organizations into either community health organizations or hospital systems.

### Analysis

We incorporated both categorical and qualitative variables into a multiple variable linear regression model for assessing our outcomes of interest: the percentage of patients achieving a suppressed HIV viral load and the percentage adhering to recommended follow-up care, defined as having an appointment every six months as recommended for clinically stable patients.^15^ This approach was chosen to account for the potential combined effects of multiple interventions or categorical variables on our outcomes. Additionally, we conducted Pearson’s coefficient tests to evaluate the association between achieving a suppressed viral load and completing medical appointments every six months.

Qualitative data emerging from the surveys, including responses to open-ended questions related to challenges and benefits of MLPs to patient population, were subjected to thematic analysis^16^ to gain deeper insights into the associations observed in our study. We examined 41 responses from legal practitioners involved with the MLPs alongside responses from clinicians, social and behavioral health service providers, and administrators. These qualitative measures aimed to explore perceived barriers and facilitators to effective MLPs in addressing gaps in the HIV care continuum, as well as perceptions regarding characteristics associated with effective MLPs.

## Results

### Sample Characteristics

Out of our 81 respondents, only 63 of them recorded percentage patients with viral load <200 and percentage of patients who completed outcome a medical appointment every 6 months. Of those 63, 3 of them did not provide information on all the variables we wanted to study. Therefore, we went ahead and completed our study with a total of 60 responses. Information regarding the categorical variables in our sample is detailed in Table 1.

**Table 1.** Summary of Characteristics of the completed MLP Surveys (n=60)

Characteristics	Number of Responses (%)
Initial Screener	
Clinician	28 (46.67%)
Law Practitioner	19 (31.67%)
Behavioral/Social Worker	18 (30%)
Administrator	2 (3.33%)
Legal Services	
On-site	26 (41.67%)
Off-site referral	25 (43.33%)
Both on-site and off-site referral	9 (15%)
Organization Type	
Hospital System	20 (33.33%)
Community Health Organization	40 (66.67%)

### Benefits

One of the greatest benefits of working in an MLP is the tendency for MLPs to have a better assessment of patient needs. Many respondents stated that MLPs provide a “more complete picture of the patient’s condition.” One respondent elaborated on this point, saying, “They seek out solutions at the individual and policy levels to a range of health-related social and legal needs, and are uniquely qualified to help the health care system disrupt the cycle of returning people to the unhealthy conditions that would otherwise bring them right back to the clinic or hospital.” Another respondent shared this sentiment, stating: “More full service, whole person treatment. Reduced barriers to care and adherence.”

A second benefit noted by respondents is that the MLP structure allows for better coordination of care services. One way MLPs provide better coordination is through strengthened communication, as illustrated by one of the respondents: “Being able to work with the staff in an open and clear manner and communicate well to resolve conflict.” Law practitioners especially were keen to share with us these benefits, as an MLP provides a “[c]lose working relationship with hospital. Easy to get medical records needed for a case. Good understanding of legal issues we can work on, etc. True partnership.” Law practitioners also found it helpful to not only receive access to medical records but also collaborate with clinicians. One respondent noted that “[b]ecause a lot of information needed for clients’ cases is medical, the partnership makes it easier to reach out to the related medical professional.” Coordination of services through strong communication and service provider collaboration establishes an environment in which wholistic patient care is easily centered.

One final perceived benefit highlighted by the respondents was that the MLP gave providers the ability to provide comprehensive, high-quality care. One respondent said that MLPs provide (as stated previously) “[m]ore full service, whole person treatment. Reduced barriers to care and adherence.” Within MLPs, patients are better connected to social workers and law practitioners, which better enables patients to adhere to their anti-retroviral treatment plan. This benefit appears to be even more robust for MLPs that integrate these services on site rather than via referral. One respondent noted that it was a benefit to have “[t]he ability to provide comprehensive care to clients instead of have to refer them to a bunch of different places.” Furthermore, one of the law practitioners remarked, “We are only there 2 out of 5 days, so if a patient does not seek referral through a provider and stop-in, they might not get assistance and never come back if we physically were not present.” Immediate access to an on-site attorney contributed to a more comprehensive care continuum for patients.

## Challenges

### Adoption Barriers

A significant perceived challenge in working in an MLP is the complexity of the process. One respondent stated that the “[p]rogram is too much,” and many others described the biggest challenge as “more steps.” What is notable about this specific line of feedback is that the respondents critiquing the adoption process tended to be physicians. Furthermore, there was some degree of distrust from the patient perspective regarding MLPs. Some providers have stated that “[p]atients are sometimes afraid to tell their immigration status for fear of being exported from the US.” One respondent said patients may completely disengage from the service noting that “[p]eople tend to avoid us to be anonymous.”

### Implementation Barriers

Another challenge shared by non-clinicians is difficulties with integrating into the healthcare system. Some respondents noted that “[m]edical providers’ policies aren’t always clear, and instead of being straightforward about whether or not they can help us, we get sent back and forth between a number of supervisors and administrators to the point where it affects our representation.” Other responses expand on this issue, with one respondent specifying that challenges include “work flow issues, physical space needs, more information about HIPAA and information sharing, figuring out what and how we want to measure outcomes … lack of full integration in medical team creates communication and training barriers … obsession with client numbers from legal organization undermines ability to focus on other factors.”

In addition to healthcare organizational barriers, there are issues with interpersonal collaboration with the healthcare team. One of the respondents identified tensions, stating that “[e]veryone wants to help the patient, but is skeptical of other service providers’ abilities to do their job. Unnecessarily hostile.”

Finally, the MLPs may have issues with managing the demands for legal counsel and needs for the patient. One of the law practitioners described this sharing, “… Legal cases take time and one lawyer cannot handle as many cases a one doctor sees patients.” This can be especially challenging in some clinical settings, such as hospitals. Another law practitioner (as previously quoted above) complained that “[h]ospital partners don’t always respect attorney client confidentiality or how time sensitive fixing problems can be.”

### Sustainability Barriers

The biggest barrier in sustaining MLP practice is the lack of sustainable funding. The difficulty of financing MLPs is a shared concern among many of the respondents of the survey. Some providers have gone so far as to say that the low amount of funding prevents them from “… properly (doing) our job.” As a result, widespread implementation of MLPs in caring for PWH has been limited.

Some respondents also have stated that the public policy barriers serve as long-term issues for MLPs in improving patient-centered outcomes. Public policy or politics can hinder the effectiveness of MLPs. One provider shared that “[t]hey are often limited in their scope. They are fantastic for medical issues but cannot help with our patients major social issues like immigration status.”

### Quantitative Trends Supported by Qualitative Findings

**Figure 1.**
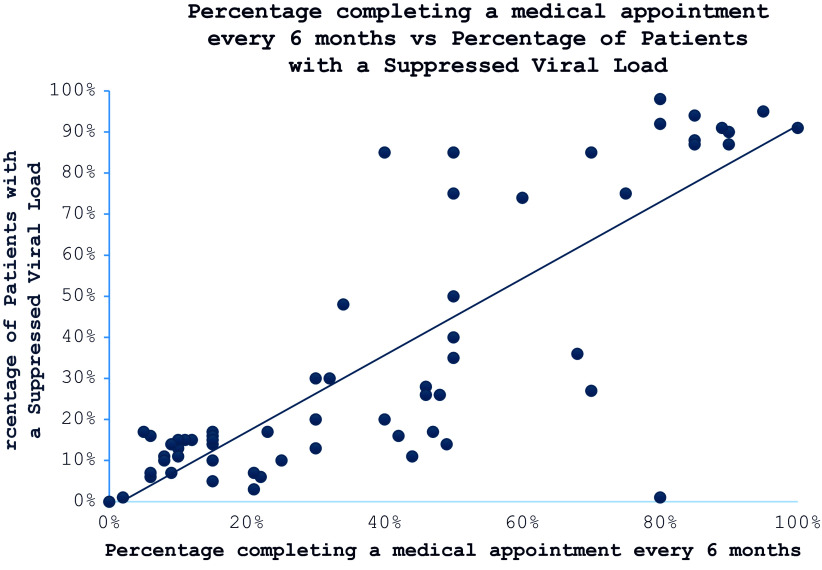
Pearson’s correlation analysis showed that percentage completing a medical appointment every 6 months was significantly and positively correlated with percentage of patients with virus load <200 copies/ml (R = 0.83, P < 0.000), suggesting that the higher the percentage completing a periodical medical appointment, the higher the percentage of patients with a low virus load.

The results of the Pearson’s correlation analysis (Figure 1) demonstrated a significant and positive correlation between the percentage completing a medical appointment every six months and the percentage of patients with viral loads <200 copies/ml (R = 0.83, p < 0.000). Conversely, having a clinician as an initial screener for health-harming legal needs showed a negative association with completing follow-up appointments every six months, as indicated in Table 2.

**Table 2.** Summary of multiple variable linear regression analysis evaluating the association of percentage completing a medical appointment every 6 months with initial screener, legal service method, number of on-site clinical services, number of referrals for a clinical service and organization type (n=60, adjusted R2 = 0.34, F = 4.33, P < 0.001)

Independent Variable	Coefficient	SE	t	P-value
Initial Screener				
Clinician	–0.19	0.09	–2.06	0.044
Law Practitioner	0.09	0.12	0.77	0.443
Behavioral/Social Worker	0.04	0.09	0.47	0.642
Administrator	–0.26	0.19	–1.34	0.187
Legal Service Method				
On-site	Ref(0)			
Off-site referral	–0.06	0.09	–0.65	0.517
Both on-site and off-site referral	–0.04	0.12	–0.3	0.762
Number of on-Site Clinical Services	0.05	0.02	3	0.004
Number of Referral to Clinical Services	0.01	0.02	0.46	0.646
Organization Type				
Hospital System	Ref(0)			
Community Health Organization	0.09	0.07	1.25	0.217
Baseline	0.19	0.1	1.82	0.074

Community-Based Organizations (CBOs) demonstrated a positive coefficient of 0.14 (p = 0.093), indicating a trend toward improved patient outcomes — specifically higher rates of viral suppression — compared to hospital systems, although the result was not statistically significant. One participant highlighted concerns about hospital-based care, stating “[h]ospital partners don’t always respect attorney-client confidentiality or understand how time-sensitive fixing problems can be.” Participant feedback shed light on various challenges faced within healthcare settings. One participant emphasized the lack of training for medical providers, stating (as quoted previously), “Lack of full integration in the medical team creates communication and training barriers.”

Moreover, our findings indicated a significant association between the presence of more on-site services and both completing follow-up appointments every six months and achieving a reduced viral load (Tables 2 and 3). Participants emphasized the critical role of on-site MLP services in patient engagement and health outcomes. One respondent underscored the importance of physical presence, stating (as quoted previously), “If a patient does not seek referral through a provider and just stops in, they might not receive assistance and may not return if we were not physically present.”

**Table 3.** Summary of multiple variable linear regression analysis evaluating the association of percentage patients with VL <200 with initial screener, legal service method, number of on-site clinical services, number of referrals to a clinical service and organization type (n = 60, adjusted R2 = 0.41, F = 5.53, P < 0.001)

Independent Variable	Coefficient	SE	t	P-value
Initial Screener				
Clinician	–0.13	0.1	–1.35	0.182
Law Practitioner	–0.02	0.12	–0.12	0.902
Behavioral/Social Worker	–0.01	0.1	–0.12	0.904
Administrator	–0.17	0.2	–0.83	0.41
Legal Service Method				
On-site	Ref(0)			
Off-site referral	–0.05	0.1	–0.49	0.629
Both on-site and off-site referral	0	0.13	–0.02	0.987
Number of on-Site Clinical Services	0.07	0.02	3.58	0.001
Number of Referral to Clinical Services	0.01	0.02	0.27	0.792
Organization Type				
Hospital System	Ref(0)			
Community Health Organization	0.14	0.08	1.71	0.093
Baseline	0.08	0.11	0.75	0.458

## Discussion

Our findings emphasize the integral structure of MLPs, showcasing the essential contributions of clinicians, lawyers, and social service providers within these innovative care models. Comparable studies have similarly recognized the significance of service integration and the distinctiveness of the MLP model in tackling health disparities.^17^ The sample predominantly comprised clinicians and law practitioners, with varying degrees of involvement from behavioral/social workers and administrators. Additionally, the distribution of legal services — whether provided on site, through off-site referral, or both — and the representation of different types of organizations, predominantly hospital systems and community health organizations, reflect the diverse nature of the MLPs surveyed.

The qualitative findings add critical contextual depth to the quantitative results, offering a nuanced understanding of patient experiences, implementation challenges, and service delivery gaps that statistical correlations alone cannot fully capture. Respondents emphasized that MLPs play a pivotal role in addressing health-related social and legal needs, enabling better care coordination, and ultimately fostering high-quality, patient-centered care. By integrating legal services into healthcare settings, MLPs help mitigate structural barriers to care, such as housing instability, discrimination, and employment-related challenges, which can significantly impact health outcomes. This support was particularly beneficial for patients, as legal assistance facilitated access to necessary resources, improved adherence to treatment plans, and reduced stressors that contribute to poor health outcomes. Additionally, respondents highlighted the effectiveness of on-site MLP services, noting that co-located legal and medical support enhances accessibility, strengthens provider-patient trust, and minimizes logistical barriers to follow-up care. The physical presence of legal professionals within healthcare settings was seen as a key factor in reducing patient attrition, streamlining referrals, and ensuring timely interventions. However, challenges such as workflow integration, communication gaps between medical and legal teams, and sustainability concerns were also noted. These insights underscore the need for continued investment in MLPs, with an emphasis on scalable implementation strategies, cross-disciplinary training, and policies that support long-term financial sustainability.

Despite the well-documented benefits of MLPs, this study identified significant challenges related to training, adoption, implementation, and sustainability. One of the primary barriers reported by respondents, particularly physicians, was the complexity of MLP processes, which made it difficult to effectively engage with legal services while managing clinical responsibilities. Without structured education on MLPs, providers may not recognize the relevance of legal interventions in addressing social determinants of health (SDoH), leading to underutilization of these critical services. Additionally, patient distrust emerged as a significant concern, particularly in communities with heightened fears surrounding immigration status, housing insecurity, or employment-related legal issues. This reluctance underscores the need for culturally and linguistically appropriate outreach strategies to foster trust and ensure that patients understand their rights and protections when engaging with MLP services.

From an implementation perspective, integrating MLPs into existing healthcare workflows presented logistical and operational challenges. Healthcare providers often operate under time constraints, limiting their ability to conduct in-depth legal screenings or facilitate referrals during patient visits. Communication gaps between medical and legal professionals further exacerbated these challenges, as differences in terminology, case management approaches, and documentation requirements can create inefficiencies. Establishing streamlined referral pathways, shared documentation systems, and interdisciplinary training can help bridge these gaps and improve MLP adoption. Concerns regarding patient confidentiality also posed a barrier to MLP implementation. Legal issues often involve sensitive personal information related to housing, employment, immigration, and family matters. Navigating privacy regulations such as the Health Insurance Portability and Accountability Act (HIPAA) while maintaining legal confidentiality standards requires careful coordination between healthcare and legal teams. Developing clear consent procedures, secure data-sharing agreements, and patient education materials on confidentiality protections will be critical to addressing these concerns.

Sustaining MLP practices over the long term was another key challenge identified in the study. Many MLPs operate with limited funding, relying heavily on grants that are often short-term and inconsistent. Without stable financial support, MLPs may struggle to maintain staff, expand services, or invest in necessary infrastructure. Furthermore, policy constraints, such as restrictions on Medicaid reimbursement for legal services, further limit financial sustainability. To address these challenges, a diversified funding approach is recommended,^18^ incorporating fee-for-service billing, hospital sponsorships, Health Resources and Services Administration (HRSA) Expanded Services supplemental funding, and continued grant support. Fee-for-service models, in which healthcare institutions reimburse legal professionals for specific services, could provide a more predictable revenue stream. Additionally, integrating MLPs into value-based care models — where reimbursement is tied to improved health outcomes — could offer another pathway for long-term sustainability. To enhance the effectiveness and longevity of MLPs, future efforts should focus on embedding legal services more seamlessly into clinical care, improving provider training, fostering patient trust, implementing sustainable financial models, and advocating for policy changes that support the integration of legal aid within healthcare settings. By addressing these barriers, MLPs can more effectively fulfill their mission of reducing health disparities and improving outcomes for vulnerable populations.

The quantitative analysis revealed several noteworthy trends that align with qualitative insights. Specifically, the positive correlation between regular medical appointment attendance and viral load suppression underscores the importance of sustained healthcare engagement in HIV management. Beyond simple attendance, retention in care is associated with improved adherence to ART, early detection and management of comorbidities, and strengthened patient-provider relationships. MLPs that enhance legal stability — such as securing housing, addressing employment discrimination, and improving access to benefits — may contribute to retention in care by alleviating stressors that often lead to disengagement from medical services. Furthermore, the finding that clinician-led health-harming legal needs screening was negatively correlated with six-month follow-up appointments suggests significant barriers to integrating legal screenings into medical workflows. Clinicians often operate within highly time-constrained visits, limiting their ability to conduct comprehensive legal screenings, address SDoH, and facilitate appropriate referrals. Additionally, the competing demands of clinical care, coupled with limited training on legal issues, may further reduce the likelihood of effective screening and intervention. Patient reluctance to disclose sensitive legal concerns in medical settings may also contribute to lower follow-up rates. Concerns about stigma, confidentiality, and potential repercussions — particularly among marginalized populations — could deter individuals from engaging in legal discussions during clinical visits. Structural barriers, such as a lack of embedded legal professionals within healthcare teams, may further impede the effectiveness of clinician-led screenings. To improve integration, future implementation strategies should explore alternative screening models that reduce clinician burden while enhancing patient engagement. Social worker-led or patient self-administered screenings — conducted through digital platforms or pre-visit intake forms — may increase uptake and follow-through by creating a more accessible and less time-intensive process. Embedding legal advocates within healthcare settings, similar to MLPs, could also facilitate streamlined referrals and enhance patient trust in addressing legal needs. Previous studies underscore the importance of training healthcare providers to recognize and address SDoH, including legal needs, within clinical practice.^19^ A structured, interprofessional approach that includes provider training, patient-centered screening models, and embedded legal resources may enhance the effectiveness of HHLN screenings, ultimately improving both healthcare engagement and legal outcomes.

CBOs demonstrated a positive coefficient, indicating a trend toward improved patient outcomes — specifically higher rates of viral load suppression — compared to hospital systems, although the finding did not reach statistical significance. This trend underscores the critical role of locally tailored service delivery models in addressing healthcare disparities and highlights the potential of CBOs to deliver more responsive and community-centered care. Unlike hospital systems, which often operate within rigid institutional frameworks, CBOs have greater flexibility in adapting services to meet the unique needs of the populations they serve. Their ability to provide culturally responsive, linguistically appropriate, and low-barrier care fosters stronger community trust and engagement, leading to increased uptake of services. Additionally, CBOs often employ peer-led outreach, mobile health services, and wraparound support, including housing, legal aid, and mental health resources, which holistically address the social determinants of health. These factors contribute to more sustained engagement in care, improved adherence to treatment, and overall better health outcomes, particularly among marginalized and underserved populations. This highlights the need to integrate CBO-driven models into broader public health and healthcare strategies to enhance equity and effectiveness in service delivery.

Moreover, the significance of on-site services in promoting appointment adherence and viral load suppression aligns with and reinforces our qualitative findings, which underscore the critical role of physical presence in the effectiveness of MLP operations. Co-locating legal and support services within clinical settings enhances trust, facilitates warm handoffs, and reduces both logistical and psychological barriers to care. On-site service delivery fosters stronger collaboration among medical and legal teams, streamlines communication, and increases the likelihood that patients will follow through with both healthcare and legal interventions. This physical integration of services plays a pivotal role in patient engagement, continuity of care, and ultimately, improved health outcomes — particularly for individuals navigating complex social and structural challenges. These findings highlight the importance of embedding co-located services within healthcare systems and MLP models as a strategy to advance health equity and maximize the impact of interdisciplinary, patient-centered care.

The findings of this study hold implications for MLPs aiming to enhance patient outcomes among PWH. Addressing the identified challenges, such as streamlining MLP processes, fostering trust, improving integration into healthcare systems, securing sustainable funding, and advocating for supportive policies, is crucial for maximizing the potential impact of MLP interventions. Future research could explore innovative strategies for overcoming these challenges, evaluate the long-term effectiveness of MLP interventions, and assess the scalability of successful MLP models across different healthcare settings and patient populations.

PWH experience unique and multifaceted barriers to care, including medical, legal, and social determinants of health that directly impact health outcomes. High levels of HIV-related stigma, medical mistrust, and structural inequalities necessitate integrated models of care that go beyond traditional healthcare settings. The implementation of MLPs within HIV care settings presents a unique opportunity to address these barriers. While the findings of this study are specific to MLPs serving PWH, the broader implications extend to MLPs serving other populations with complex medical and social needs, such as individuals with other chronic conditions who face similar barriers to care.

## Limitations

The study has several limitations. First, our study was limited by a small sample size and incomplete responses, which impacted the statistical power and generalizability of our findings. Of the 81 responses received, 17 had to be excluded due to missing data on key variables, further reducing our analytical sample. A limited sample size can restrict the ability to detect meaningful associations and may introduce potential biases in data interpretation. Additionally, as a cross-sectional study, we were unable to establish causal relationships between our variables of interest. Future research should prioritize larger, more diverse samples and longitudinal designs to enhance the validity, applicability, and causal inferences of the findings. Since our study was cross-sectional, we could not fully assess causality for our variables of interest. Second, we chose to group various on-site or referral clinical or social services as one quantitative variable, which assumes each type of service counted has an equal effect on viral load suppression and completing follow-up appointments. Future studies should measure the individual effectiveness of each type of service in relation to our outcomes of interest. Third, we acknowledge the limitations of not having clearly defined variables such as screener role, legal service model, and organization type. Additionally, other factors that may influence MLP implementation, such as legal problem type and levels of legal service, were not analyzed in this study. Future research should address these gaps. Fourth, since this was a voluntary survey, there could have been some degree of response bias where people with the strongest opinions are more likely to respond, skewing the results. Finally, we selected these outcomes based on the HIV care continuum due to their well-documented association with long-term morbidity and mortality. Future research should explore additional health indicators, such as mental health status and stress reduction, to provide a more comprehensive understanding of the factors influencing HIV care and overall well-being.

## Conclusion

Study findings underscore the nuanced relationship between various factors impacting the care outcomes of individuals with HIV. Findings related to a decrease in follow-up appointments when screenings for HHLN were conducted by clinicians underscores the need for comprehensive training among clinicians. The differential impact of community-based organizations compared to hospital settings on reducing viral loads highlights the significance of community integration in improving HIV care continuum outcomes for PWH. This emphasizes the potential advantages of community-driven healthcare initiatives and practices and the need for further exploration and uptake of these practices. Additionally, the association between increased on-site services and improved outcomes — both with engagement in care and viral load suppression — underscores the pivotal role of comprehensive, accessible services encompassing clinical, social, legal, and behavioral healthcare. Participants highlighted the importance of on-site legal services, stressing the importance of one-stop shop models of care. Future studies should delve deeper into identifying the specific, on-site services that yield the most substantial influence on enhancing outcomes for individuals with HIV, facilitating more targeted and effective care strategies.

## Data Availability

The datasets generated and/or analyzed during the current study are not publicly available due to the sensitivity of the data but are available from the corresponding author on reasonable request.
